# Antimicrobial Nano Coatings

**DOI:** 10.3390/nano12234338

**Published:** 2022-12-06

**Authors:** Angela Ivask, Merja Ahonen, Karin Kogermann

**Affiliations:** 1 Institute of Molecular and Cell Biology, University of Tartu, 51010 Tartu, Estonia; 2 Faculty of Technology and Research Center WANDER, Satakunta University of Applied Sciences, FI-26101 Rauma, Finland; 3 Institute of Pharmacy, University of Tartu, 50411 Tartu, Estonia

History has demonstrated that the uncontrolled fast thriving of potentially pathogenic microorganisms may lead to serious consequences and, thus, the approaches helping to control the microbial numbers in infectional hot-spots are necessary. From the point of view of public health, strict control over microbial spread is necessary in medical practice, healthcare facilities, as well as in crowded public areas. Since the 1940s, antibiotics have been traditionally used to control microbial numbers and spread in medical practice [1]. However, the spreading of antibiotic resistance is currently calling for caution in antibiotics overuse and effective alternative solutions are sought [2]. With the recent progress in nanotechnology and advancement of nanomaterial-directed research [3], the potential of nanosize, i.e., 1–100 nm, particles and structures in various applications has been realized. Due to their small size, high surface reactivity, and multiple mechanisms of action, nanoparticles, and structures have been proven themselves also as effective antimicrobials [4]. 

Surfaces play a central role in transmission of microbes [5] and, thus, decreasing the number of microorganisms on inanimate surfaces has been a focus in a series of research articles, as well as a target for many commercial antimicrobial products. The increased popularity of nanotechnology has also resulted in elevated use of nanoparticles and nanostructures in and on antimicrobial surfaces. The most often used nanostructures are spherical nanoparticles but also nanotubes, nanowires, nanofibres, and more exotic forms of nanopillars and nanoflakes have been used for that purpose [6] (Figure 1). Those different types of nanostructures can be grown or deposited onto the surface, or woven into textiles or filters. Chemically, the most often used antimicrobial nanostructures are composed of silver followed by chitosan, antimicrobial peptides, zinc, copper, titanium, and others (Figure 1). The surfaces are usually either simple depositions of nanomaterials, embedded nanomaterials, or elongated structures, such as nanofibers, nanowires, nanotubes, nanopillars, nanoneedles, or even bio-inspired structures [7] (Figure 1). Among the target organisms which inhibition is measured on antimicrobial surfaces are bacteria, fungi, yeasts, but also viruses. A 2018 study by Rosenberg et al. [8] demonstrated that a significant proportion of antimicrobial surfaces concerned antifouling and antibacterial surfaces. Articles on antifungal surfaces were clearly in minority compared with antibacterial studies and articles on antiviral surfaces were the lowest in quantity. One must certainly acknowledge that with the appearance of COVID-19 pandemic, the share of antiviral articles has significantly increased. According to Clarivate Web of Science, in 2021 and 2022 the amount of articles on “antiviral” increased by 36 and 44% compared with five earlier years. Additionally, the current Special Issue on antimicrobial nanomaterials includes articles on antiviral activity of nanomaterials.

The areas of application where antimicrobial surfaces have been most commonly used include external surfaces, such as marine surfaces, textiles, paints, food-related surfaces and medical devices, but also internal surfaces to the human body, such as catheters and implants [8]. Due to this variety of application areas, also the spectrum of target organisms for antimicrobial coatings is relatively wide. However, due to recommendations by a variety of antibacterial testing standards as well as availability of laboratory test strains, the clearly prevailing test microorganisms have been *Escherichia coli* and *Staphylococcus aureus* [8]. Other bacteria, even clinically more relevant, have been in clear minority. Interestingly, the inhibition of microbial biofilms by antimicrobial surfaces have only been mentioned rarely and mostly in the context of catheters [8], despite their importance in the spread of a wide variety of infections and abundance on most environmental surfaces [9].

This Special Issue on antimicrobial nanocoatings includes articles on antimicrobial nanomaterials applied onto hard carriers or surfaces [10,11], onto the surfaces of catheters [12] or woven into wound dressing materials [13,14]. The types of nanomaterials that were discussed in these articles include nanoparticles or nanostructures of Ag, CuO, ZnO, ZnO/Ag [10,11] or ZnO/Au [15] nanocomposites, antimicrobial peptides [12], natural plant-derived antimicrobial compounds [13] and antibiotics [14]. Only one article, Dediu et al. [15] assessed the efficacy of their proposed antibacterial agent in a suspension assay, without surface deposition. In other papers, the methods used for surface application of nanomaterials included thin film creation on hard carrier [11], embedding into acrylic matrix [10], sonochemical deposition [12], electrospinning [13,14], and crosslinking of an antimicrobial agent to hydrogel materials [14], thus, in most cases creating stable antimicrobially active surfaces. Indeed, the stability of antimicrobial surfaces is one of the key properties that should be studied prior their real-life usage. For most surfaces, a slow release of active agents is preferred, to extend and maximise their antimicrobial activity. In line with that, Rosenberg et al. [10] showed that leaching of Zn or Ag ions from surfaces covered with acrylic polymer-embedded ZnO/Ag was negligible as the release of both Ag and Zn was very low. It was shown that over 10 simulated use cycles those surfaces remained intact, i.e., there were no signs of visible degradation of the surfaces. Ivanova et al. [12] demonstrated the stability of antimicrobial peptide polymyxin B loaded polymethacrylate coatings during 7 days under simulated real usage of urinary catheters. On the other hand, electrospun *Chelidonium majus* plant extract loaded Polycaprolactone/Polyvinyl Alcohol_Pectin nanofibres meant for use in wound dressings, were expected to release the active antimicrobial, as well as degrade during usage [13]. Thus, as appropriate for transient and frequently changed wound dressings, the activity time and self life of such fibres is expected to be relatively short. Analogously, Martin et al. [14] demonstrated more than 80% release of tetracycline from electrospun fiber-reinforced hydrogels within 1 h, to ensure a fast and effective antibacterial action upon application of such materials onto the wounds.

In addition to the physical stability, an important feature of antimicrobial surfaces is also safety to non-target organisms and in human use. Certainly, the closest contact with end-users occurs in case of internal surfaces and so, biocompatibility analysis of polymyxin-loaded antimicrobial catheters [12] and *C. majus* extract containing wound dressings [13] demonstrated their good compatibility with human cells and tissues. Martin et al. [14] found that their tetracycline-containing hydrogels affected to some extent also the human cells and, thus, suggested further optimization of such hydrogel scaffolds.

The efficacy of antimicrobial surfaces described in this Special Issue was in most cases analysed against bacteria. The most traditional species, *Escherichia coli*, *Staphylococcus aureus* [10,14,15], and *Pseudomonas aeruginosa* [12,13], mostly in planktonic form, but also as biofilms [13]. None of the articles used fungi or yeasts for efficacy testing, but Merkl et al. [11] used viruses as targets for antimicrobial compounds in thin films. A variety of methods were used to study the effects of antimicrobial coatings to bacteria, starting from initial efficacy screening of the antimicrobial compounds in a tetrazolium/formazan assay [15], microdilution assay [13] till real application imitating assessment of the efficacy of wound dressings in a semi-solid agar matrix [13,14]. Application-relevant testing conditions were applied also for antibacterial testing of plywood surfaces covered with acrylic matrix embedded ZnO/Ag nanocomposites by Rosenberg et al. [10]. In this paper, in addition to the traditional ISO 22196 test, where a thin liquid layer of bacterial cultures is exposed to a surface, bacterial exposure to surface in droplets at variable relative humidities (RH) was used. Bacteria proved to be more sensitive to ZnO/Ag composite-based surfaces at higher RH levels while dry conditions decreased the antibacterial activity of those surfaces. Even more closer to the application-relevant testing was the use of an in vitro model to assess the formation of *P. aeruginosa* biofilms on different regions of the polymyxin B-treated catheter of the human bladder and constantly supplied with artificial urine, to assess the formation of *Pseudomonas aeruginosa* biofilms on different regions of the catheter [12]. According to the results, the applied antimicrobial coating was equally effective in catheter tip and within the balloon. Importantly, to claim an antibacterial effect, certain efficacy conditions should be fulfilled. According to US EPA interim guidance, supplemental residual antimicrobial products, such as coatings and films, should exhibit at least 3 log reduction in bacterial CFU within at least 2 h [16] and according to European legislation, at least 3 log decrease in CFU is required during 60 min [17]. Those requirements were generally met in most of the papers of this Special Issue. According to Rosenberg et al. [10], wooden surfaces covered with acrylic matrix embedded ZnO/Ag nanoparticles decreased the CFU of *E. coli* and *S. aureus* by >2.8 and >2.8 logs, respectively, within 2 h. Catheter surfaces treated with zwitterionic polymer combined with polymyxin B decreased the attachment and growth of *P. aeruginosa* by 8 logs within 24 h [12]. Parallel experiments with *P. aeruginosa* biofilms showed a 97% decrease in biofilm formation on those catheters. Nanofibrous wound dressings supplemented with antibacterial *C. majus* extract decreased the CFU counts of *S. aureus* by 3.82 logs [13]. The efficacy of those wound dressings towards *P. aeruginosa* was however slightly lower and reached only 1.32 logs. Similarly, the study on antiviral activity of ZnO, CuO, and Ag nanomaterials by Merkl et al. [11] did not reach the officially required 3 logs decrease in viral plaque forming units, but showed around 1 log (90%) decrease in SARS-CoV-2 PFU counts. The latter result obtained for Ag nanoparticle containing surfaces is however a promising result from a research perspective. The paper by Dediu et al. [15] that tested the efficacy of ZnO/Au nanomaterials in suspension before their application to any surfaces demonstrated ~65 and 75% decrease in viability of *E. coli* and *S. aureus* within 3 h of exposure, which may not be sufficient to fulfill the requirements for antibacterial claims but shows the potential of Au nanoparticles as adjuvants.

In conclusion, the papers collected in this Special Issue reflect the existing widespread interest in synthesis, characterization, efficacy analysis, and potential applications of nanomaterial- or nanostructure-based antimicrobial coatings. Although the Special Issue cannot fully cover the topic of antimicrobial nanomaterial-based surface coatings, we are confident that its contributions will open new perspectives and bring innovation to the field of antimicrobial materials and relevant products.

## Figures and Tables

**Figure 1:**
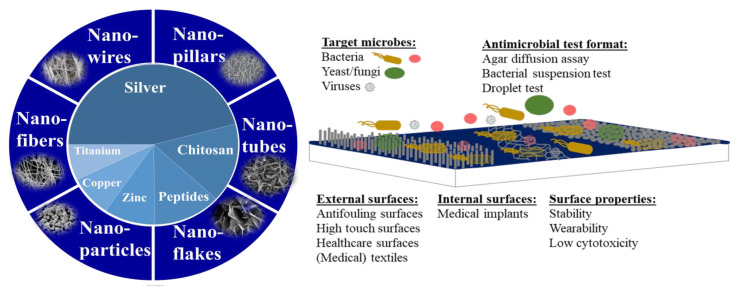
Forms of antimicrobial nanomaterials and the key properties of nanomaterial-based antimicrobial surfaces. Left side: antimicrobial agents based on literature search in ISI WoS (January 2022) when out of 33,000 articles matching keywords “antimicrob* AND nano*”, 12,552 were on silver, 4400 on chitosan, 3519 on peptides, 2663 on zinc, 2272 on copper, and 1975 on titanium. The physical forms of antimicrobial nanomaterials include elongated nanostructures, such as nanofibres, -wires, -pillars, or nanotubes, nanoparticles or other structures. Right side: nanoparticle, nanopillar or nanofiber structures deposited or embedded onto antimicrobial surfaces. Common surface types, key physico-chemical properties, microbial targets, and antimicrobial testing formats are shown.
